# Supplementary material for: Binding of the periplakin linker requires vimentin acidic residues D176 and E187
Source: Commun Biol. 2020 Feb 21;3:83. doi: 10.1038/s42003-020-0810-y (PMC7035337; doi:10.1038/s42003-020-0810-y)
Supplement: Supplementary file 1 — Supplementary Information [file 42003_2020_810_MOESM1_ESM.pdf]

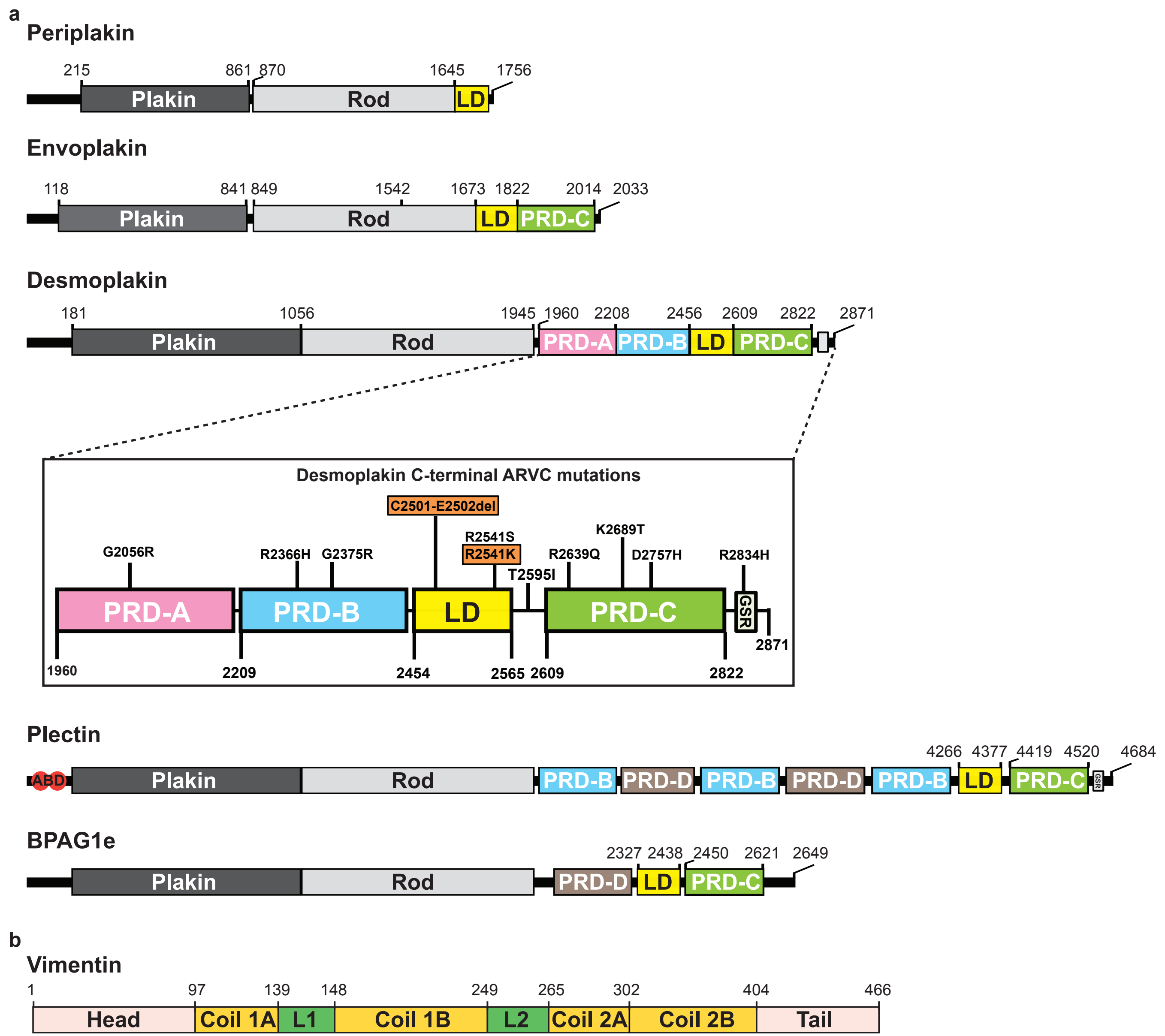

Supplementary Figure 1. Domain architecture of plakin family members periplakin, envoplakin, desmoplakin, plectin and BPAG1e, and the intermediate filament protein vimentin. a) All three plakin proteins possess an N-terminal plakin domain and a central rod domain that forms a helical coiled-coil. The C-terminal tail domain of periplakin consists solely of a linker domain (LD). The equivalent region of envoplakin is comprised of a LD and a plakin repeat domain (PRD-C), whereas desmoplakin exhibits a LD, three PRDs (PRD-A, B and C) and a glycine-serine-arginine (GSR)-rich region. The domain boundaries are indicated. The boxes contain close-ups highlighting the positions of some pathogenic mutations that have been identified in the desmoplakin C-terminal tail region. All result in an ARVC-like phenotype[1] except R2366H which causes skin fragility-woolly hair syndrome[2]. ARVC mutations that were characterised in this study are highlighted (orange boxes). ABD, actin binding domain. b) Vimentin encompasses a highly α-helical central domain (residues K97-L404), with flanking non-helical 'head' and 'tail' modules.

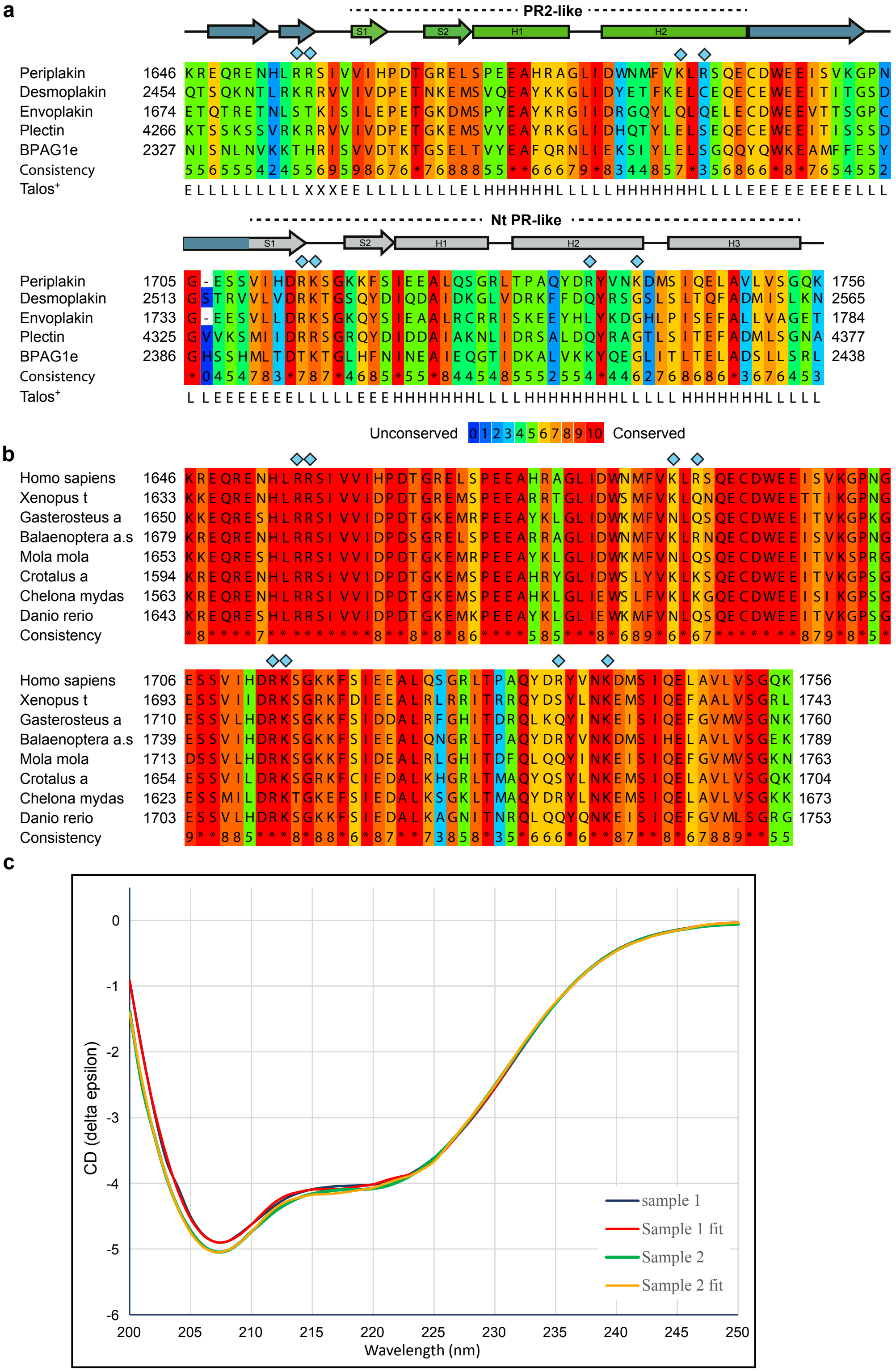
a
PR2-like
S1
S2
H1
H2
Periplakin 1646
Desmoplakin 2454
Envoplakin 1674
Plectin 4266
BPAG1e 2327
Consistency
Talos+
Nt PR-like
S1
S2
H1
H2
H3
Periplakin 1705 1756
Desmoplakin 2513 2565
Envoplakin 1733 1784
Plectin 4325 4377
BPAG1e 2386 2438
Consistency
Talos+
Unconserved 0 1 2 3 4 5 6 7 8 9 10 Conserved
b
Homo sapiens 1646
Xenopus t 1633
Gasterosteus a 1650
Balaenoptera a.s 1679
Mola mola 1653
Crotalus a 1594
Chelona mydas 1563
Danio rerio 1643
Consistency
Homo sapiens 1706 1756
Xenopus t 1693 1743
Gasterosteus a 1710 1760
Balaenoptera a.s 1739 1789
Mola mola 1713 1763
Crotalus a 1654 1704
Chelona mydas 1623 1673
Danio rerio 1703 1753
Consistency
c
CD (delta epsilon)
Wavelength (nm)
sample 1
Sample 1 fit
Sample 2
Sample 2 fit

Supplementary Figure 2. Sequence alignments of plakin protein linker domains and circular dichroism spectra of the periplakin linker protein. a) Alignment of linker domain sequences from human plakin proteins. Sequences were obtained from Uniprot (accession numbers O60437 (periplakin), P15924 (desmoplakin), Q92817 (periplakin), Q15149 (plectin) and Q03001-3 (BPAG1e)). Alignment was performed using the PRALINE multiple sequence alignment toolkit[3]with the colour scheme showing the degree of amino acid conservation. The secondary structure based on the crystal structure of the periplakin linker domain is shown above the sequence alignment (strand (arrow), helix (rectangle) and coil (black solid line)). The colour coding is as shown in Fig 1a. Also shown are the secondary structure elements of the desmoplakin linker domain predicted by Talos+ (E=strand; H=helix; L=loop and X=no assignment). b) Alignment of linker domain sequences from different species. Sequences were obtained from Uniprot (accession numbers O60437 (homo sapiens), F7DLC3 (Xenopus tropicalis), G3NKG0 (Gasterosteus aculeatus), A0A452CGT3 (Balaenoptera acutorostrata scammoni), A0A3Q3WJP8 (Mola mola), A0A0F7Z4V1 (Crotalus adamanteus), M7BNQ5 (Chelona mydas) and A0A0R4IAS2 (Danio rerio)). Positively charged periplakin linker domain that were mutated as part of this study are highlighted (blue diamonds). c) Circular dichroism spectra of the periplakin linker protein. Two independent samples (22.1 μM and 38.4 μM) in 5mM Hepes (pH7.5), 50 mM NaCl were analysed. Secondary structure content was estimated using BeStSel (http://bestsel.elte.hu)[4]: 31% α-helix, 29% β-sheet and turn and 40% other secondary structures.

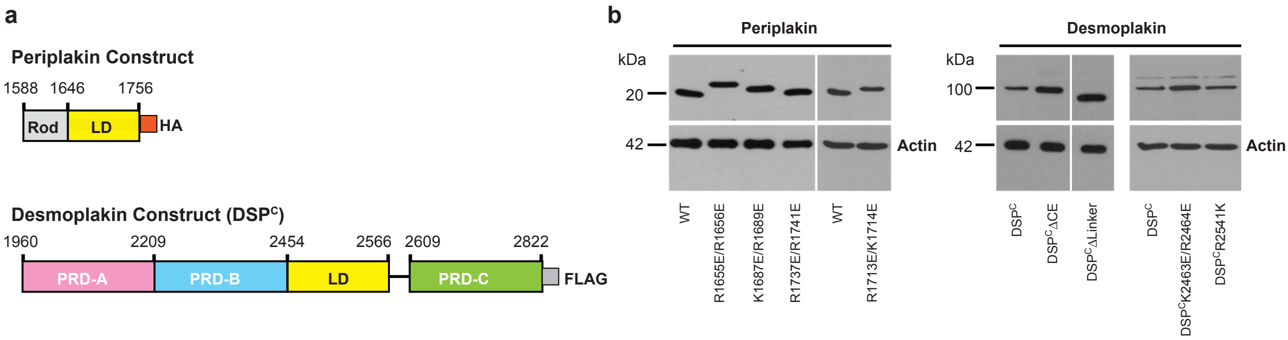

Supplementary Figure 3. Periplakin and envoplakin constructs used in transfection experiments. a) Periplakin and desmoplakin constructs used to transfect HeLa cells. b) Western blots showing expression of periplakin and desmoplakin wild-type and mutant proteins in transfected HeLa cells. Periplakin and desmoplakin proteins were detected using anti-HA and anti-FLAG antibodies respectively.

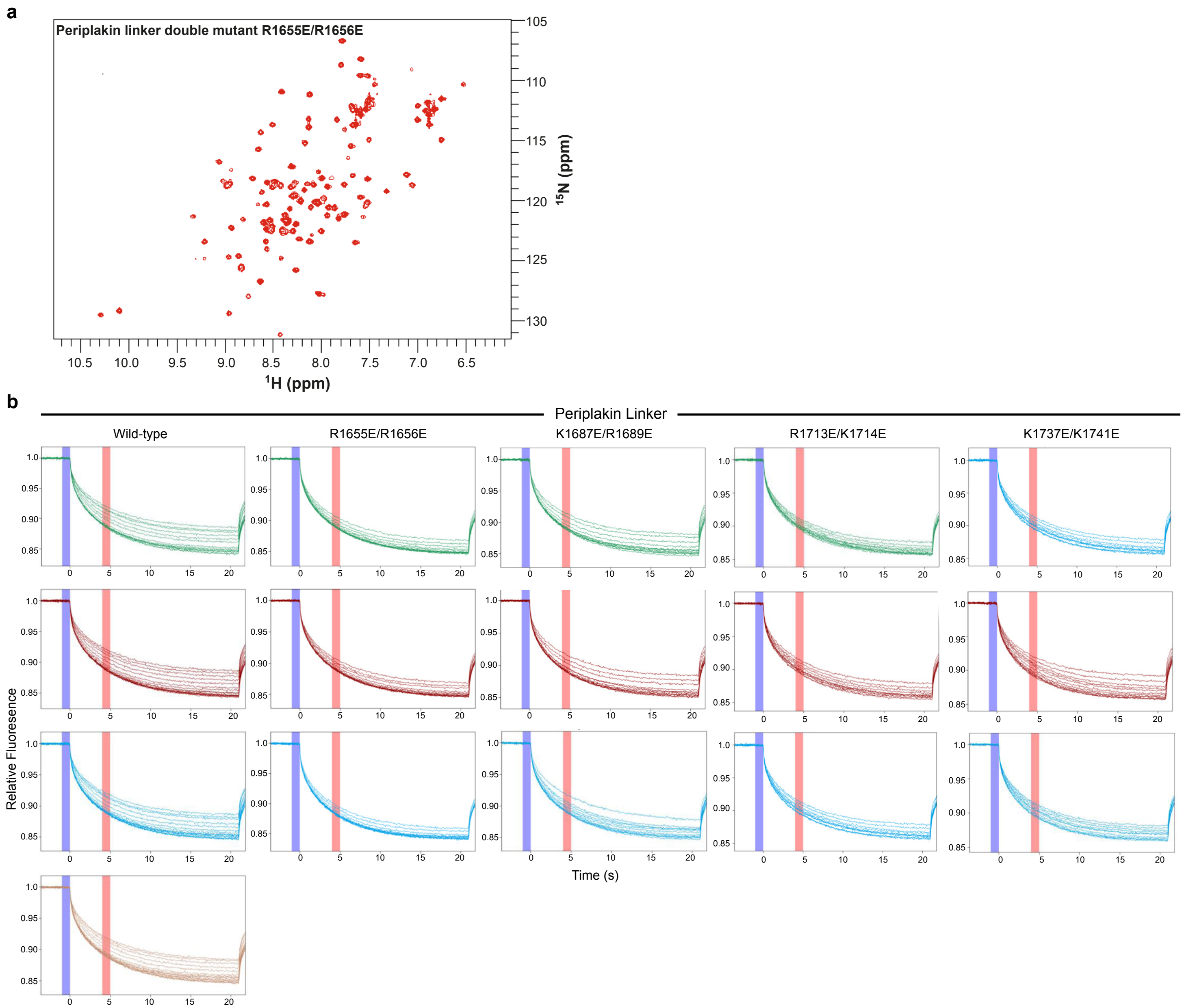

Supplementary Figure 4. HSQC spectrum of periplakin linker mutant R1655E-R1656E and MST traces showing binding of periplakin linker proteins to the vimentin^{ROD}. a) HSQC spectrum of periplakin linker double mutant R1655E/R1656E, showing that the mutant protein is correctly folded. Periplakin mutants K1687E/R1689E, R1713E/K1714E and R1737E/K1741E showed similar spectra.
b) MST raw data traces from binding experiments with the indicated measured MST-on time (blue line F0 and pink lines F1). The data were analysed and the results shown in Fig.2a. The binding constants and fit statistics for each data set are shown in Supplementary Table 1.

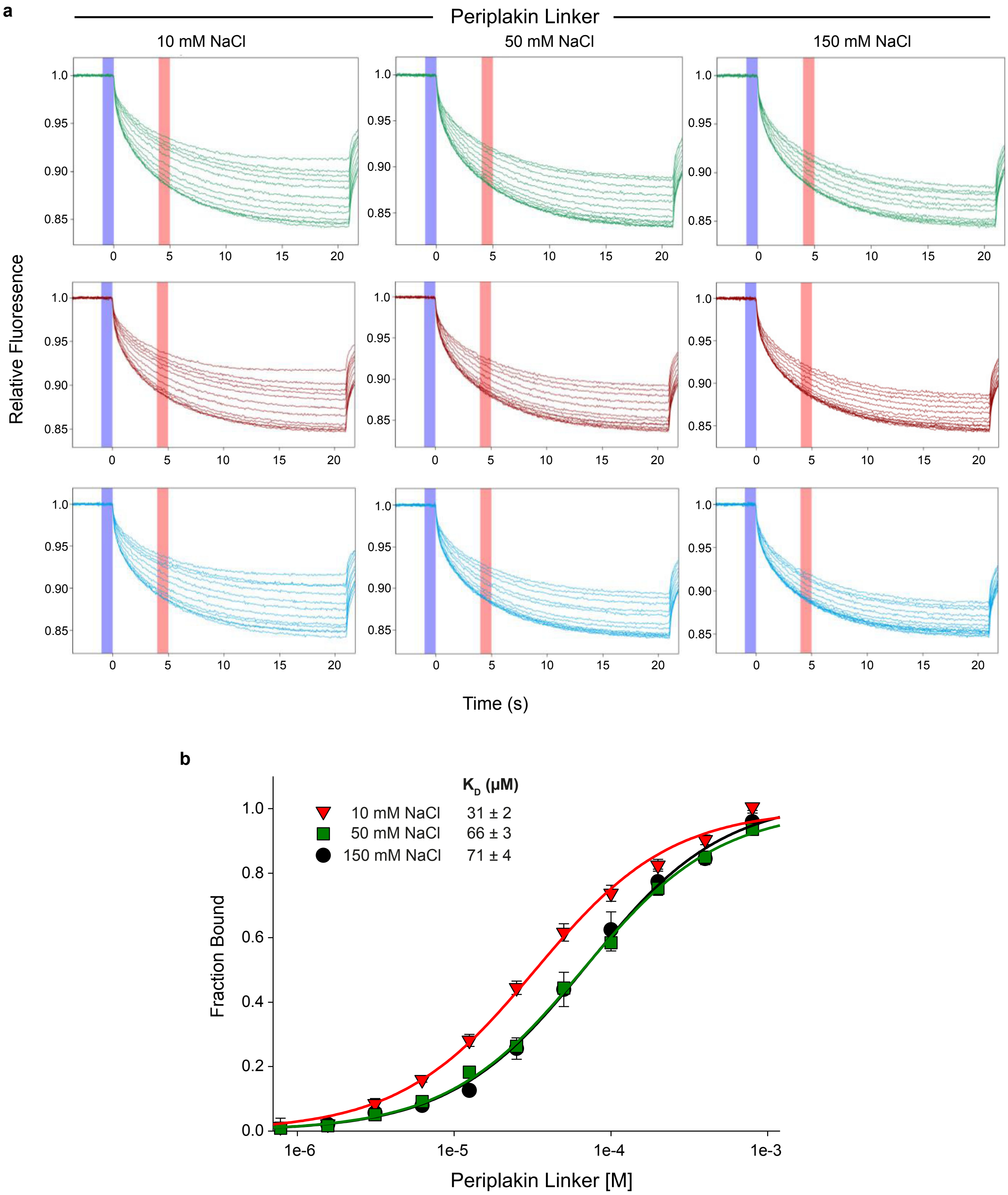

Supplementary Figure 5. Effect of salt on binding of the periplakin linker to vimentin^{ROD}. a) MST raw data traces from binding of the periplakin linker to vimentin^{ROD} at 10, 50 and 150mM NaCl. The MST-on time used for data analysis was 5 seconds. b) The periplakin linker binds to the vimentin^{ROD} with a higher affinity at low salt concentration. The binding constants and fit statistics are listed in Supplementary Table 1.

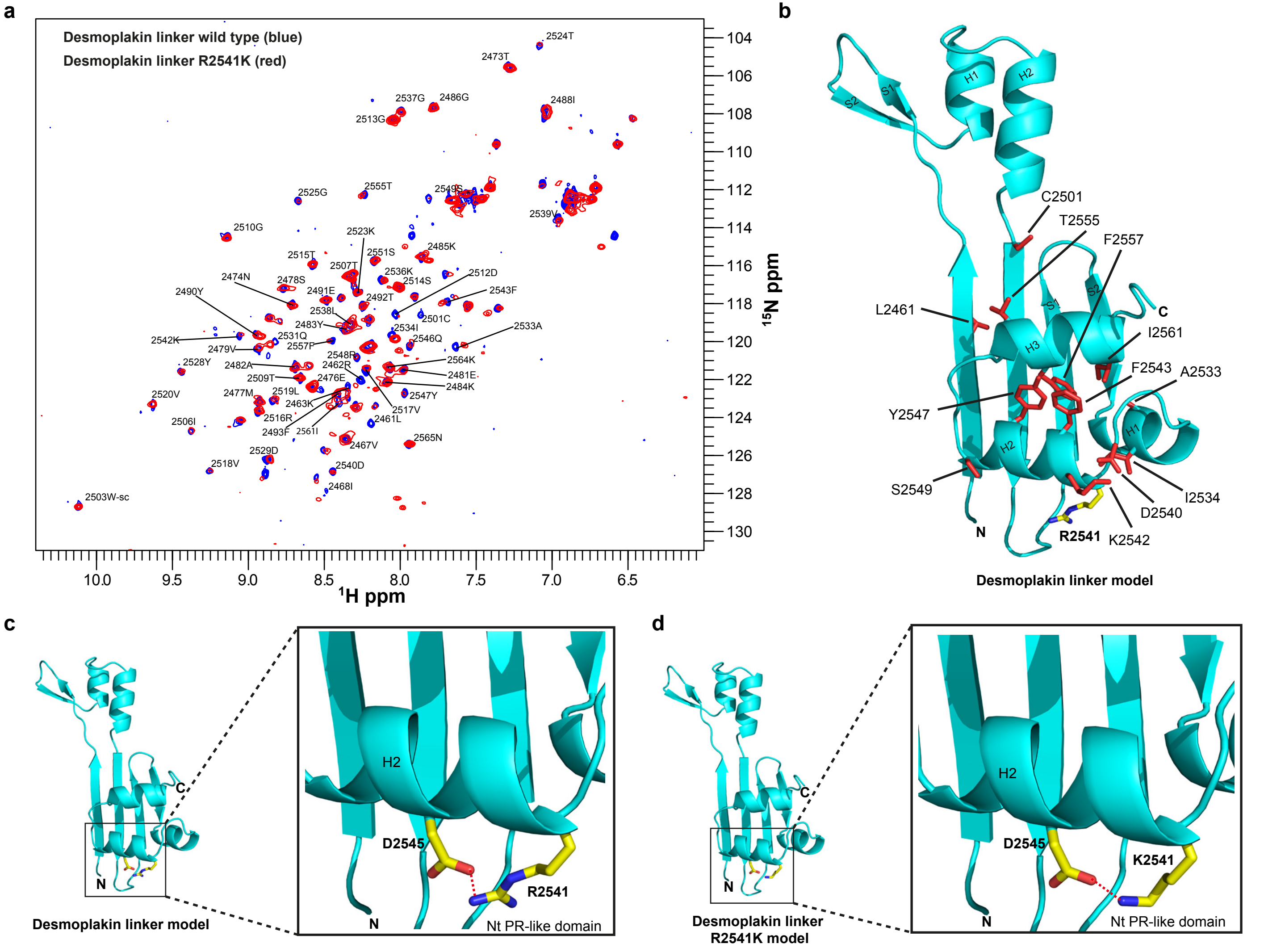

Supplementary Figure 6. ARVC mutation R2541K causes only minor perturbations in desmoplakin linker domain structure.
a) Superimposed two dimensional 1H,15N-resolved NMR spectra of wild-type (blue) and ARVC R2541K mutant (red) desmoplakin linker proteins. b) Mapping chemical shift perturbations between the wild-type and R2541K desmoplakin linker proteins onto the desmoplakin linker model structure. Residues with the largest chemical shift perturbations are shown (red). c) Ribbon representation for desmoplakin linker model showing the intra salt bridge interaction (red dashed line) between residues R2541 and D2545.
Box highlights close up views of the relevant interactions. d) Ribbon representation for desmoplakin linker model highlighting the putative compensatory ionic bonding (red dashed line) between K2541 and D2545 in the R2541K variant form. Box highlights close up views of the relevant interactions.

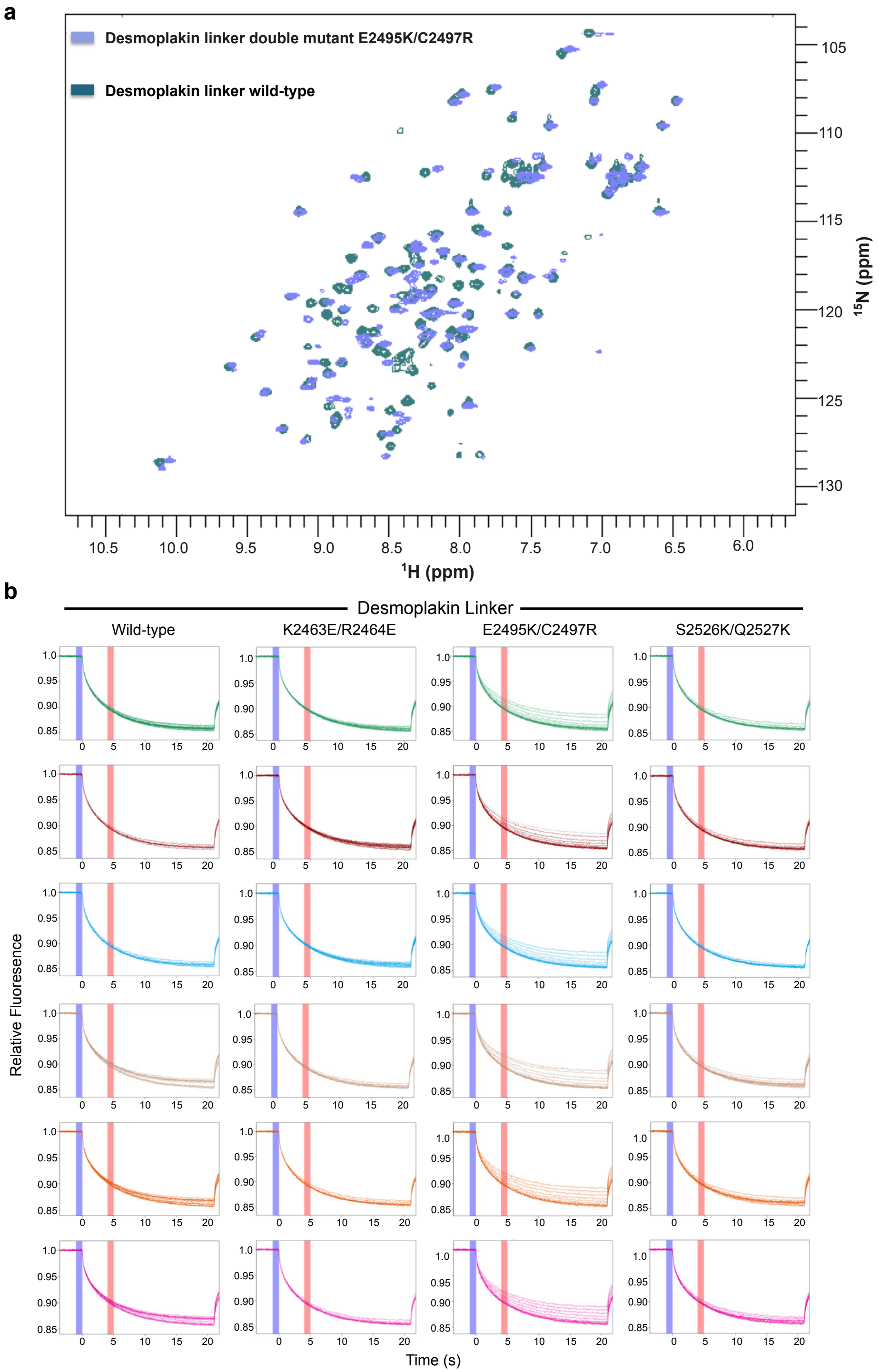

Supplementary Figure 7. HSQC spectra of desmoplakin linker mutant E2495K/C2497R and MST traces showing binding of desmoplakin linker proteins to the vimentin^{ROD}. a) Overlay of the HSQC spectrum of desmoplakin linker domain double mutant E2495K-C2497K (light-blue) and desmoplakin linker wild type (teal) showing that the mutant protein is correctly folded. Desmoplakin mutants K2463E/R2464E and S2526K/Q2527K showed similar spectra. b) A total of six data sets were collected for each experiment and analysed using a 5 second MST-on time. The binding and fit statistics are listed in Supplementary Table 2 and the fits are shown in Fig. 5b.

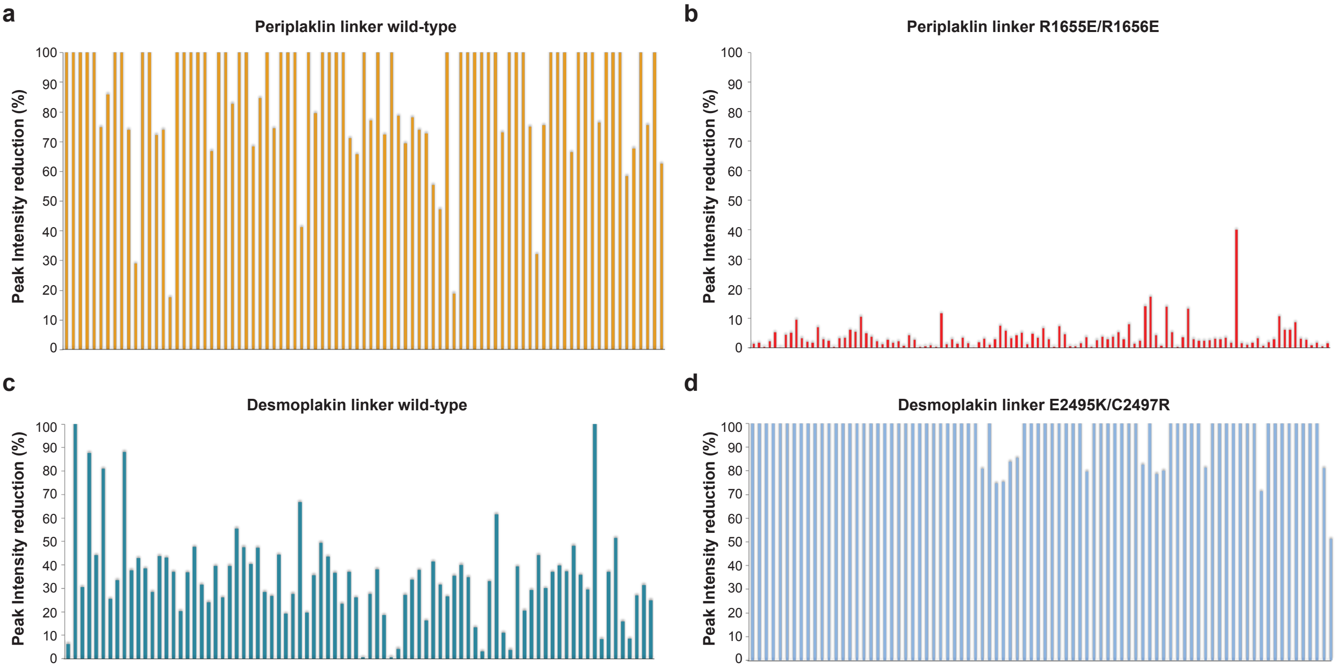

Supplementary Figure 8. Histograms showing reduction in peak intensity in ^{15}N-HSQC spectrums of periplakin and desmoplakin linker proteins following the addition of vimentin. Vimentin^{FL} (200 μM) was added to ^{15}N-labelled linker proteins (100 μM) in the absence of salt. Addition of vimentin to wild-type periplakin linker resulted in only a small number of peaks retaining 20% of their starting peak intensity. In the case of the wild-type desmoplakin linker a greater number of peaks retained intensity, indicating weaker binding. The majority of peaks showed little loss of intensity following the addition of vimentin to periplakin linker mutant R1655E/R1656E, indicating lack of interaction between the two proteins. By contrast the majority of peaks showed significant loss of intensity following addition of vimentin to desmoplakin mutant E2495K/C2497R, indicating enhanced interactions.

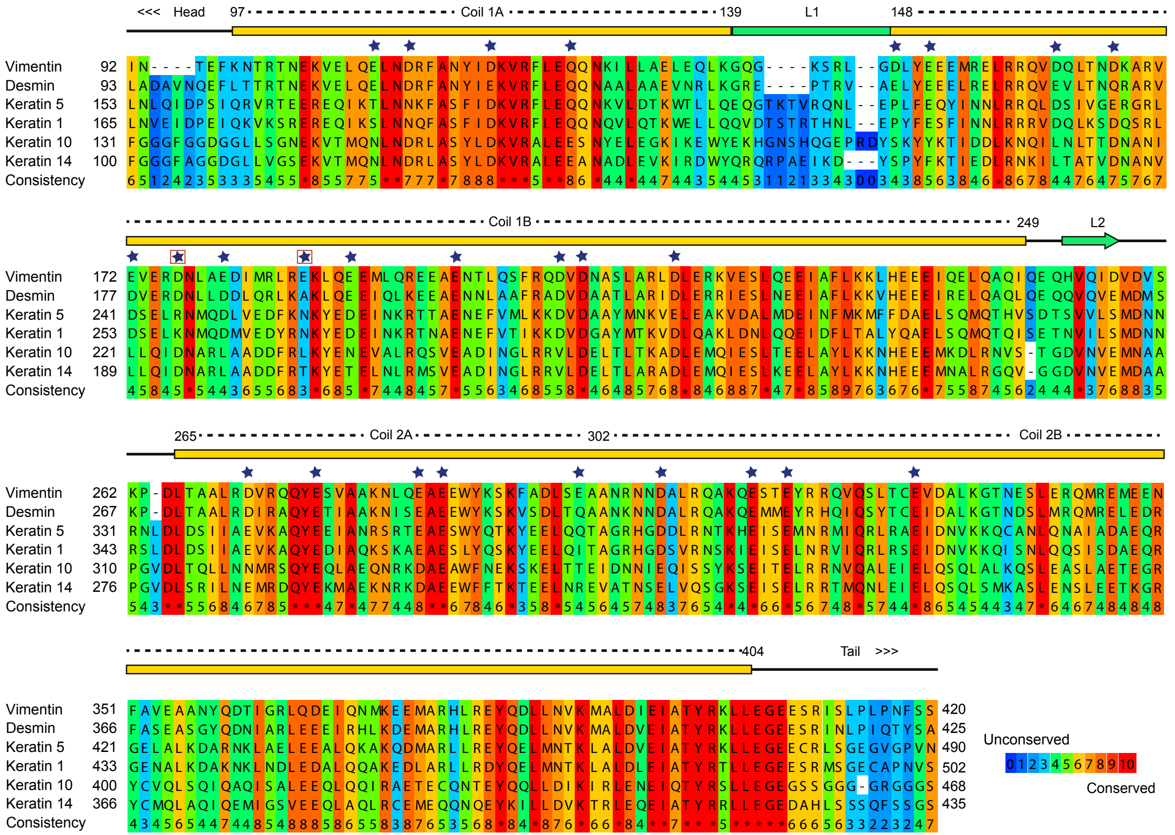

Supplementary Figure 9. Sequence alignment of intermediate filament proteins. Human intermediate filament protein sequences were obtained from Uniprot (accession numbers P08670 (vimentin), P17661 (desmin), P13647 (keratin 5), P04264 (keratin 1), P13645 (keratin 10) and P02533 (keratin 14)) and aligned using the PRALINE program. The colour scheme shows the degree of amino acid conservation. Negatively charged vimentin residues that were selected as restraints for docking studies with the periplakin linker are highlighted (blue stars). Vimentin residues that are important for binding to the periplakin linker domain based on our MST data are also shown (red square boxes). The secondary structure based on the crystal structure of available vimentin fragments is shown above the sequence alignment (strand (arrow), helix (rectangle) and coil (black solid line).

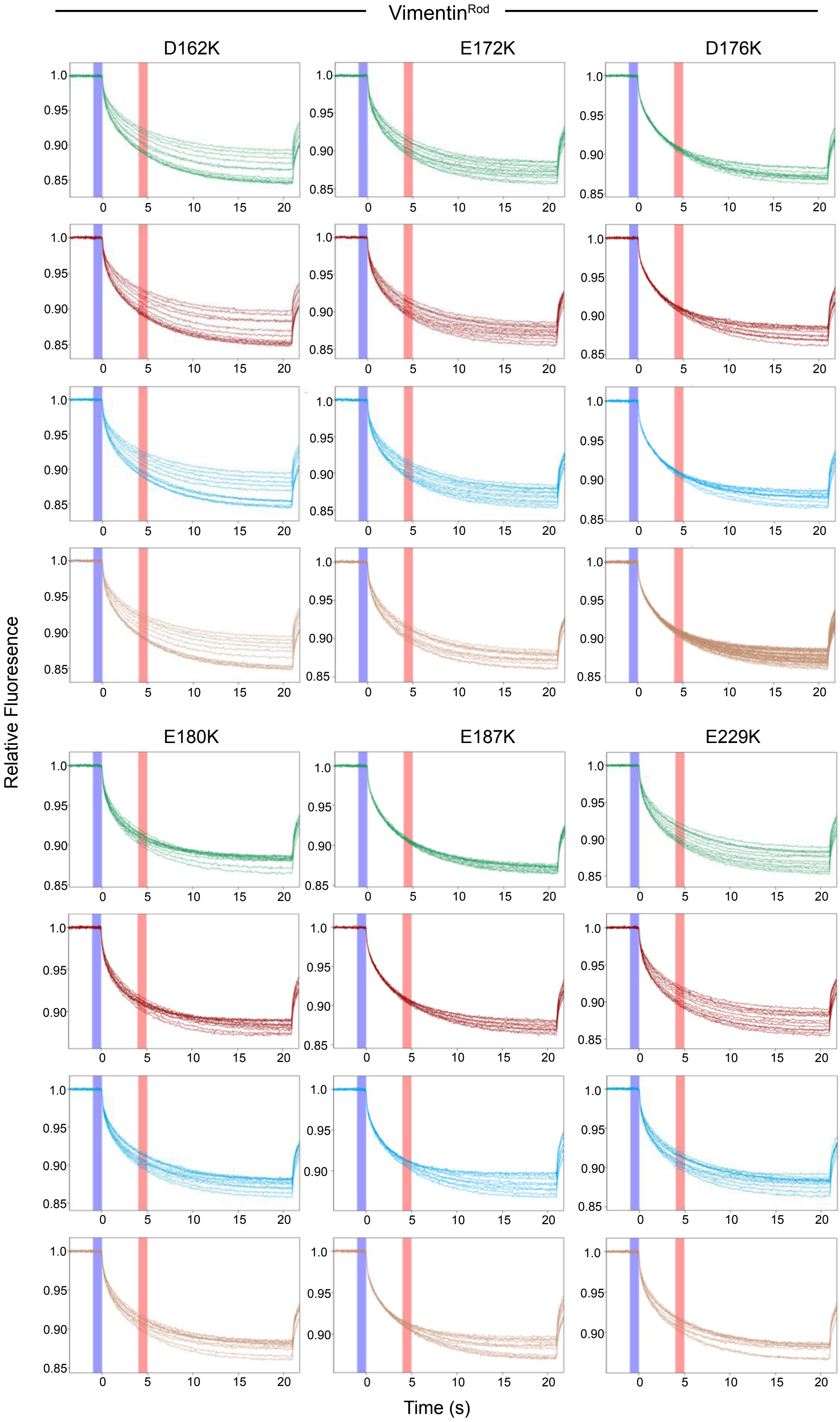

Supplementary Figure 10. MST traces showing binding of the periplakin linker to vimentin^{ROD} mutants. The raw data traces are shown with the MST-on time of 5 seconds used for analyses indicated. The binding constants and fit statistics are listed in Supplementary Table 4 and the fits are shown in Fig.6c.

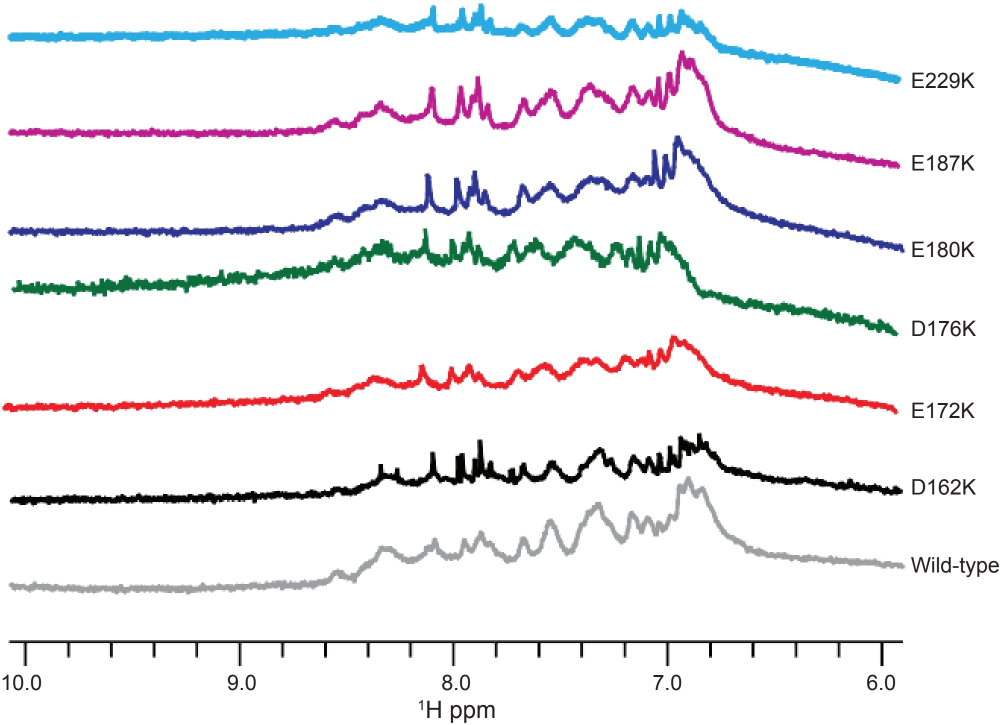

Supplementary Figure 11. Vimentin^{ROD} mutants are correctly folded. The 1H-NMR spectra of each of the vimentin proteins are shown. The backbone amide region from 6.5 ppm to 9.0 ppm shows well dispersed sharp peaks, which confirms the presence of well-folded protein. The peak distribution among the mutants is similar based on the 1D proton spectra and confirms that the mutations did not introduce a change in protein conformation.

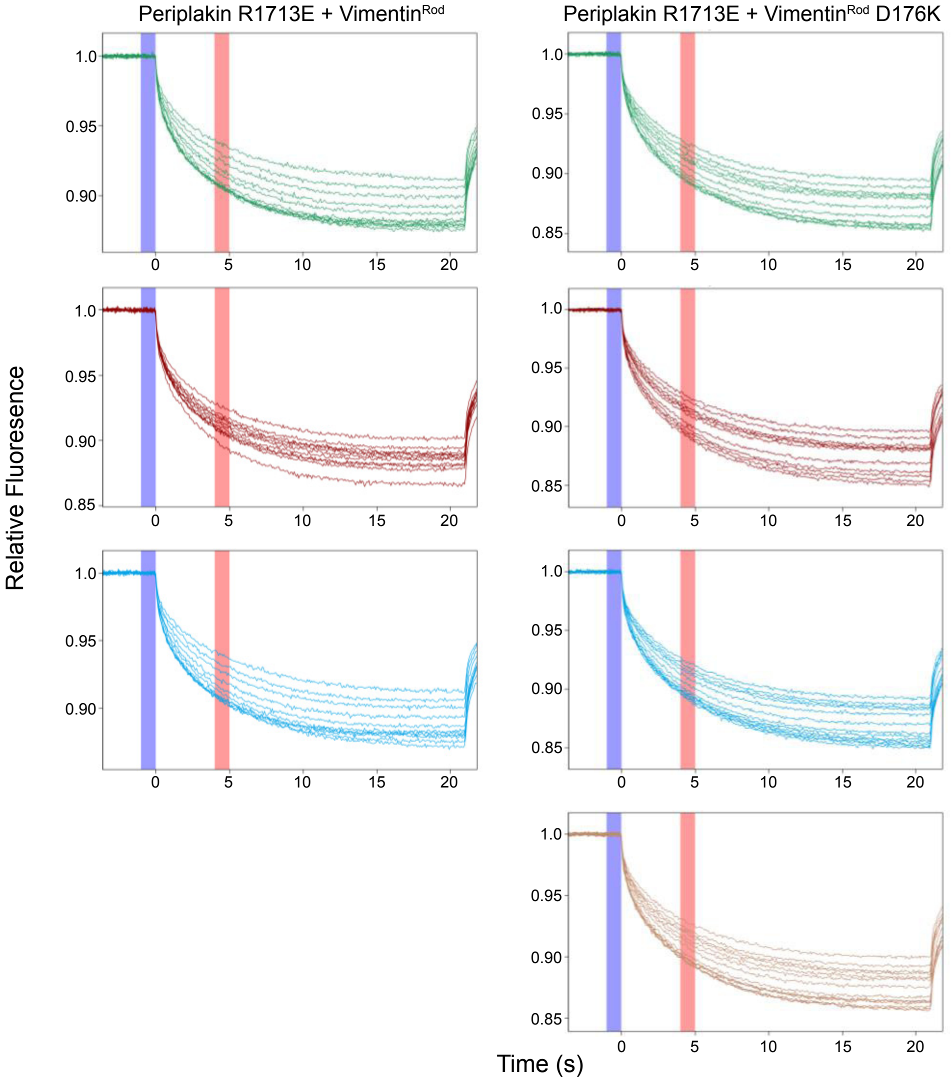

Supplementary Figure 12. MST traces showing binding of periplakin linker mutant R1714E to wild-type and D176K vimentin^{ROD}. The raw data traces are shown with the MST-on time of 5 seconds used for data analysis. The binding constants and fit statistics are listed in Supplementary Table 4 and the fits are shown in Fig.6d.

Supplementary Table 1. Summary of fits for periplakin linker binding to vimentin^{ROD} by MST

Vimentin^{ROD}	Periplakin Linker	Assay	K_D μM	n	S
Wild-type	Wild-type	1	59 ± 6	1	0.8
	150mM NaCl	2	66 ± 12	1	0.5
		3	70 ± 12	1	0.4
		4	85 ± 24	1	0.7
		Merged	70.5 ± 3.8	4	0.4
Wild-type	Wild-type	1	59 ± 2	1	0.6
	50mM NaCl	2	69 ± 4	1	0.6
		3	71 ± 5	1	0.8
		Merged	66.0 ± 3.2	3	0.6
Wild-type	Wild-type	1	31 ± 3	1	1.1
	10mM NaCl	2	31 ± 3	1	1.3
		3	32 ± 2	1	0.9
		Merged	30.2 ± 2.3	3	1.2
Wild-type	R1655E/R1656E	1	351 ± 18	1	0.5
		2	382 ± 21	1	0.5
		3	444 ± 32	1	0.6
		Merged	380 ± 51	3	0.6
Wild-type	K1687E/R1689E	1	312 ± 15	1	0.7
		2	306 ± 21	1	1.0
		3	273 ± 87	1	1.7
		Merged	300 ± 54	3	1.1
Wild-type	R1713E/K1714E	1	143 ± 27	1	1.5
		2	145 ± 42	1	1.4
		3	123 ± 24	1	0.8
		Merged	135 ± 30	3	1.1
Wild-type	R1737E/K1741E	1	57 ± 13	1	1.8
		2	43 ± 9	1	1.6
		3	29 ± 5	1	1.4
		Merged	47.9 ± 11	3	1.9

The MST experimental data from individual experiments or merged data sets was fit to a one-ligand binding model to determine the binding affinity K_D with an error of one standard deviation. Binding of periplakin linker mutants to wild-type vimentin^{ROD} was performed in 150mM NaCl. The number of experiments (n) and the goodness of fit is shown as S, the standard error of regression.

Supplementary Table 2. Summary of fits for desmoplakin linker binding to vimentin^{ROD} by MST

Vimentin^{ROD}	Desmoplakin Linker	Assay	K_D μM	n	S
Wild-type	Wild-type		No binding	6	
Wild-type	K2463E/R2464E		No binding	6	
Wild-type	E2495K/C2497R	1	620 ± 100	1	0.7
		2	760 ± 200	1	1.1
		3	619 ± 130	1	0.9
		4	680 ± 100	1	0.8
		5	632 ± 85	1	0.6
		6	437 ± 83	1	1.0
		Merged	600 ± 67	6	0.6
Wild-type	S2526K/Q2527K		No binding	6	

The MST experimental data from individual experiments or merged data sets was fit to a one-ligand binding model to determine the binding affinity K_D with an error of one standard deviation. The number of experiments (n) and the goodness of fit is shown as S, the standard error of regression.

Supplementary Table 3. Summary of docking results for periplakin linker and different vimentin structures using HADDOCK

Vimentin PDB code	Vimentin Fragment	Haddock score	Cluster size	Z-score	RVE (kcal/mol)	Overall RMSD (Å)	BSA (Å^2)	Vimentin restraints
3S4R	T99-L189	-119.7 ± 16.3	7	-1.6	141.9 ± 32.65	4.3 ± 0.1	1908.3 ± 71.9	D148, E151, D162 and D167
3SWK	E153-H238	-119.5 ± 6.3	23	-1.7	42.2 +/- 32.49	13.3 +/- 0.3	1408.5 +/- 111.2	E172, D176, E180 and E187
3UF1	L146-I249	-119.1 ± 7.1	22	-2.3	109.5 ± 40.12	17.6 ± 0.2	1803.7 ± 172.7	E191, E200, D209, D211 and D219
3KLT	D264-K334	-115 ± 2.5	22	-1.8	90.9 ± 18.4	3.5 ± 0.2	1264.3 ± 101.7	D271, E277, E286 and E288
3G1E	N102-L138	-104.6 ± 8.7	7	-1.8	100 ± 18.94	1.1 ± 0.7	1273.8 ± 156.3	E109, D112, D119 and Q126
3KLT	D264-K334	-102.2 ± 6.7	11	-1.4	58.5 ± 36.82	14.3 ± 0.6	1460.8 ± 105.5	E300, D307, E315, E318, E329

Haddock statistics are listed for the top cluster of each periplakin linker-vimentin model. RMSD is the root mean square deviation from the overall lowest energy structure. Conserved vimentin residues selected for ambiguous intermolecular restraints are also shown. RVE, restraints violation energy; BSA, buried surface area. Z-score indicates how many standard deviations from the average this cluster is located in terms of score.

Supplementary Table 4. Summary of fits for periplakin linker binding to vimentin^{ROD} by MST

Vimentin^{ROD}	Periplakin Linker	Assay	K_D µM	n	S
D162K	Wild-type	1	71 ± 10	1	1.4
		2	56 ± 4	1	0.6
		3	76 ± 5	1	0.6
		4	66 ± 7	1	1.0
		Merged	67.8 ± 3.2	4	0.6
E172K	Wild-type	1	26 ± 5	1	1.2
		2	24 ± 7	1	1.4
		3	27 ±8	1	1.6
		4	29 ± 6	1	1.3
		Merged	27.0 ± 4.4	4	1.2
D176K	Wild-type		No binding	4	
E180K	Wild-type	1	8 ± 2	1	1.5
		2	4 ± 2	1	1.7
		3	4 ± 2	1	1.9
		4	3 ± 2	1	1.7
		Merged	5.7 ± 2.4	4	1.7
E187K	Wild-type		No binding	4	
E229K	Wild-type	1	12 ± 3	1	1.4
		2	8 ± 3	1	1.5
		3	27 ± 5	1	1.3
		4	25 ± 3	1	1.0
		Merged	16.4 ± 2.4	4	1.7
Wild-type	R1713E	1	210 ± 26	1	0.9
		2	160 ± 21	1	1.1
		3	240 ± 91	1	0.7
		Merged	200 ± 29	3	0.8
D176K	R1713E	1	20 ± 6	1	2.2
		2	22 ± 5	1	2.2
		3	28 ± 5	1	2.0
		4	24 ± 4	1	1.8
		Merged	23.1 ± 3.7	4	1.5

The MST experimental data from individual experiments or merged data sets was fit to a one-ligand binding model to determine the binding affinity K_D with an error of one standard deviation. The number of experiments (n) and the goodness of fit is shown as S, the standard error of regression.

Supplementary Table 5. PCR primers used to construct periplakin and desmoplakin plasmids for expression in cultured HeLa cells and bacteria

Construct	Primer
Periplakin M1588-K1756 in pcDNA3.1(-) (with HA tag)	AATCTAGAATGGCAGCGACGGAAACACGAG (*Xba*I)
	TTAAGCTTCTAGGCGTAGTCAGGCACGTCGTAAGGATACTTCTGCCCAGATACCAAGACC (*Hind*III)
Desmoplakin T1960-A2822 in pcDNA3.1(-) (with Flag Tag)	TTGGTACCATGACCGTTGACACCTCCAAGCTG (*Kpn*I)
	TTCTCGAGCTACTTATCGTCGTCATCCTTGTAATCAGCCGAAGACATGTTGTAAGGG (*Xho*I)
Periplakin K1646-K1756 in pGEX-6P-1	AAGGATCCAAGCGGGAGCAGCGGGAG (*Bam*HI)
	AAGAATTCTCACTACTTCTGCCCAGATACCAA (*Eco*RI)
Desmoplakin Q2454-N2565 in pGEX-6P-1	AAGGATCCCAGACATCACAAAAGAATACC (*Bam*HI)
	TTGAATTCCTAATTTTTCAAGGAGATCATGTC (*Eco*RI)

Restriction sites used for cloning are underlined and indicated in parentheses. Vimentin constructs were produced as described[5]. All mutants were produced using the QuikChange Lightening site directed mutagenesis kit (Agilent). Mutagenic primers were designed as described by the manufacturers. HA, haemagglutinin antigen.

Supplementary References

1. van der Zwaag PA, et al. A genetic variants database for arrhythmogenic right ventricular dysplasia/cardiomyopathy. Hum Mutat 2009; 30:1278-1283.

2. Al-Owain M, et al. Novel homozygous mutation in DSP causing skin fragility-woolly hair syndrome: report of a large family and review of the desmoplakin-related phenotypes. Clin Genet 2011; 80:50-58.

3. Bawono P, Heringa J. PRALINE: a versatile multiple sequence alignment toolkit. Methods Mol Biol 2014; 1079:245-62.

4. Micsonai A et al. BeStSel: a web server for accurate protein secondary structure prediction and fold recognition from the circular dichroism spectra. Nucleic Acids Res 2018; 46:W315-W322.

5. Fogl C et al. Mechanism of intermediate filament recognition by plakin repeat domains revealed by envoplakin targeting of vimentin. Nat commun 2016; 7:10827.
